# Estimating the scale of hospital admissions for people experiencing homelessness in England: a population-based multiple systems estimation study using national Hospital Episode Statistics

**DOI:** 10.1136/bmjph-2025-002978

**Published:** 2025-10-28

**Authors:** Serena April Luchenski, Dankmar Böhning, Robert Aldridge, Fiona Stevenson, Shema Tariq, Andrew C Hayward

**Affiliations:** 1 Institute of Epidemiology and Healthcare, University College London, London, UK; 2 S3RI & Mathematical Sciences, University of Southampton, Southampton, UK; 3 Institute of Health Informatics, University College London, London, UK; 4 Institute for Health Metrics and Evaluation, University of Washington, Seattle, Washington, USA; 5 Institute of Global Health, University College London, London, UK; 6 UK Health Security Agency, London, UK

**Keywords:** Ill-Housed Persons, Health Services Accessibility, Population Surveillance

## Abstract

**Background:**

People experiencing homelessness have substantial health needs and poor access to primary healthcare, resulting in high rates of hospital care. Housing status is not routinely recorded in English electronic health records, undermining service planning. We developed methods to estimate the scale of hospital admissions for people experiencing homelessness in England.

**Methods:**

We analysed admissions for people experiencing homelessness using Hospital Episode Statistics for 2013/2014, 2015/2016 and 2017/2018. We applied multiple systems estimation Poisson regression methods to estimate total admissions and an inflation factor to correct for under-reporting. We calculated unadjusted admission rates per 1000 population per year and admission rate ratios compared with the housed population.

**Results:**

We observed 34 790 admissions in 2017/2018, with total homeless admissions estimated at 176 342 (95% CI 164 031 to 188 654) (inflation factor=5.07 (95% CI 4.71 to 5.42)). The unadjusted admission rate for the 2017/2018 homeless population was 879.0 admissions per 1000 population per year (95% CI 817.7 to 940.4), 2.5 (95% CI 2.3 to 2.7) times higher than the housed population. Restricted to rough sleepers and hostel residents, the unadjusted rate was 3516.7 per 1000 (95% CI 3271.2 to 3762.2), with a rate ratio of 10.0 (95% CI 9.3 to 10.7) compared with the housed population.

**Conclusions:**

We estimated five times as many hospital admissions for people experiencing homelessness than we observed directly. We advise caution when applying these inflation factors to other datasets because of methodological limitations in this study and sensitivities to local coding practices. In the absence of routine housing status recording, multiple systems estimation could facilitate improved service planning.

**WHAT IS ALREADY KNOWN ON THIS TOPIC**

- People experiencing homelessness have some of the worst health outcomes in society, but the scale of healthcare needs is poorly understood because of the lack of routine recording of housing status in electronic health records.

**WHAT THIS STUDY ADDS**

- We applied a new set of code lists to identify hospital admissions for people experiencing homelessness and used multiple systems estimation (also known as capture–recapture estimation) to quantify and adjust for underreporting within English hospital data. We showed that there were approximately five times more admissions for people experiencing homelessness than we could observe directly in national data, but there were important methodological limitations which necessitate a tempered interpretation of the findings.

**HOW THIS STUDY MIGHT AFFECT RESEARCH, PRACTICE OR POLICY**

- This study provides valuable insights into the potential scale of hospitalisation for people experiencing homelessness in England and a recommended set of methods for further investigating this problem. While the magnitude of underestimation should be interpreted cautiously, this study contributes important evidence for developing effective policies and services that can meet the needs of this underserved population.

## Introduction

 Homelessness is not solely defined by rooflessness or literal homelessness, but also includes individuals living in insecure, unstable or inadequate housing situations.^1^ The transient and hidden nature of this population makes it notoriously difficult to measure.^2^,^4^ The Crisis Homelessness Monitor^4^ models annual estimates of ‘core homelessness’ in England, which includes rough sleepers, people living in unconventional accommodation (eg, squatting), hostels, unsuitable temporary accommodation (eg, bed and breakfasts) and sofa surfing (eg, staying with non-family, on a short-term basis, in overcrowded conditions). There were an estimated 242 000 core homeless households in England in 2022, up from 221 000 in 2020, 224 000 in 2018 and 206 000 in 2012.^4^

People experiencing homelessness often have complex health needs, including intersecting physical illness, mental illness and alcohol and drug use disorders.^5^ It has been estimated that nearly a third of deaths among people experiencing homelessness in England are amenable to timely and effective healthcare, compared with about a quarter in the most deprived areas of the general population.^6^ The all-cause standardised mortality ratio has been estimated to be 7.88 (95% CI 7.03 to 8.74) higher than the general population for men and 11.86 (95% CI 10.42 to 13.30) higher for women.^7^

This population encounters multiple barriers to accessing primary care and preventative services in the community and often only use healthcare in a crisis.^38^,^12^ This results in higher levels of emergency department attendances, and emergency admissions and readmissions to hospital,^3 13 14^ than the general population. In turn, this contributes to poor health outcomes and high healthcare costs.^1315^,^17^

Estimating the magnitude of homelessness-related inpatient activity is fundamental to assessing the need for and impact of specialist and mainstream health and social care services for this underserved population. However, housing status is not systematically collected in English NHS health services.^13 18^ Moreover, despite there being an ICD-10 code for homelessness, coding of homelessness within routine hospital data is inconsistent.

Prior to this study, assessment of hospital care utilisation of people experiencing homelessness in England has often relied on the ‘No Fixed Abode’ (NFA) code^1317^,^19^ as a proxy for single people sleeping rough or in a hostel. This approach has notable limitations, including underascertainment (some may give a temporary address), misclassification of people who are not experiencing homelessness but decline to give an address (eg, fearing disclosure of treatment such as termination of pregnancy), and misclassification due to poor data quality or errors. Other research has examined the hospital records of people identified through specialist homeless healthcare services.^514 20^,^22^ However, sampling through specialist services introduces selection biases in terms of who is referred or attends services. There are also surveys of hospital care utilisation among people experiencing homelessness.^23^ Such surveys are also prone to selection biases in who responds or not as well as measurement bias because they rely on self-report of healthcare utilisation.

We aimed to develop a novel and robust methodology using multiple systems estimation (MSE) to overcome these limitations and estimate the scale of hospital admissions among people experiencing homelessness in England. The specific objectives were:

1. Accurately estimate the number of hospital admissions for people experiencing homelessness in England, addressing underreporting in routine hospital data.
2. Quantify the healthcare utilisation of the homeless population by calculating the rate of hospital admissions per 1000 people and compare it to the housed population.

## Methods

### Study design

This research employed a population-based, repeated cross-sectional study design, using anonymised hospital records from England’s Hospital Episodes Statistics (HES) for the fiscal years 2013/2014, 2015/2016 and 2017/2018. The analysis focused on ‘continuous inpatient spells’, which represent complete hospital admissions, accounting for transfers between different hospital providers within a single admission. These spells were identified using unique patient identifiers (HESID).

### Participants and setting

The study population encompassed all publicly funded National Health Service (NHS) inpatient hospitals in England, including acute mental health trusts. This broad inclusion ensured that the findings were representative of the national healthcare system. Individuals aged 18 years and older who were experiencing homelessness during their hospital admission constituted the target population.

Housing status is not routinely recorded within English hospital data, so we developed a homelessness phenotype to more accurately identify people experiencing homelessness within the HES data.^24^ The phenotype is described in full in the Health Data Research UK Phenotype Library^24^ and includes:

1. NFA: address recorded as NFA, with certain exclusions as per previous research^13^
2. HGP (homeless general practitioner): registered at a known HGP practice that exclusively serves those experiencing homelessness (as mapped in a previous study^25^). We used this existing list from this study to produce a corresponding list of GP practice codes using the NHS Digital ODS Portal^26^ to identify registered people within HES.
3. Z59.0: a diagnosis that includes the International Classification of Diseases version 10 (ICD-10) code for homelessness (Z59.0), usually as a secondary diagnostic code.

Our phenotype builds on previous studies which have relied on NFA alone^13 27^ to identify people experiencing homelessness. Although we have not done a formal validation study, it is highly likely, however, that the phenotype still underestimates homelessness because there are no national policies on homelessness coding and practices will vary from hospital to hospital. For example, a service evaluation showed that 58%–75% of a known hospitalised homeless population were identifiable using a similar phenotype to ours.^28^ It is this unknown level of underestimation at a national level which is the rationale for conducting this study using MSE.

### Data access and ethical considerations

Data access was facilitated through the UCL Institute of Health Informatics’ secure Data Safe Haven. This environment provided a secure and controlled setting for the analysis of sensitive patient data, ensuring compliance with data protection regulations. The UCL Institute of Health Informatics held a full copy of HES for the duration of this study. Data access was granted conditional on a data sharing agreement, consistent with prior agreements with NHS Digital. Ethical approval for this study was granted by the UCL Research Ethics Committee (Ref: 11607/001). The study used anonymised data, ensuring that individual patient identities were protected throughout the research process.

### Outcomes

The primary outcome of this study was the observed and estimated frequencies of hospital admissions per year for people experiencing homelessness in England, addressing the known underreporting issue. Secondary outcomes included the estimated admission rate per 1000 population per year, providing a standardised measure of healthcare utilisation, and the overall admission rate ratio comparing the homeless population to the housed population, highlighting potential disparities in hospital use.

### Analysis

The analytical approach aimed to estimate the true prevalence of hospital admissions among people experiencing homelessness in England, accounting for underreporting in routine hospital data, and to quantify their healthcare utilisation. Analyses were conducted using STATA V.17.

#### Data preparation and cleaning

Continuous inpatient spells, representing complete hospital admissions, were constructed using established data cleaning rules from the University of York Centre for Health Economics and the Department of Health. Unique patient identifiers (HESID) were used to link episodes of care within a single admission, ensuring accurate representation of admission events. The predefined homelessness phenotype was applied to the cleaned data.

#### Descriptive analysis

Descriptive statistics were generated to characterise the observed hospital inpatient activity. Frequencies of admissions were calculated overall and stratified by each homelessness code (NFA, HGP, Z59.0). The overlap between admissions identified by different codes was visualised to illustrate the extent of co-occurrence. Basic demographic data (age, sex and ethnicity) were calculated for both the housed and homeless populations within the dataset, providing context for potential demographic influences on hospital utilisation.

#### Multiple systems estimation

To estimate the true number of hospital admissions among people experiencing homelessness, MSE was employed. Each homelessness code (NFA, HGP, Z59.0) was treated as a separate ‘list’. Log-linear (Poisson) regression was used to model the dependence structure between these lists, accounting for potential correlations. Eight possible models were fitted, varying in the inclusion of two-way interaction terms between the lists. The Bayesian information criterion (BIC) was used to assess model fit. Due to the wide range of estimates produced for the unobserved population, Bayesian model averaging was conducted. This involved calculating weighted averages of the three best-fitting models (those with the lowest BIC) to produce more stable estimates of the total number of admissions and corresponding SEs. A final 95% CI was computed based on the normal distribution. An inflation factor was calculated by dividing the MSE-estimated total admissions by the observed total admissions, providing an estimate of the true scale of homeless in-patient activity.

To ensure the validity of our MSE, we addressed the four core assumptions: population closure within a single admission, accurate matching between lists using HESID, independence of lists and homogeneous capture probabilities. While the first three assumptions were adequately met, the fourth, concerning homogeneous capture probabilities, presented a challenge. Ideally, this assumption would be addressed by incorporating covariates that might influence the likelihood of an individual being identified by each homelessness code. However, the low rate of duplicate coding across lists (specifically, 11.6% of admissions with two codes and 0.5% with all three) precluded the inclusion of covariates. This limitation arose from the potential for numerical instability that would accompany stratification of the data with such sparse overlap. Consequently, we proceeded with the MSE without covariate adjustment, acknowledging this constraint in our discussion of study limitations. For a detailed discussion on the four assumptions and how they were met (or not), please consult the analysis section of the online supplemental appendix.

#### Estimation of admission rates and rate ratios

To quantify healthcare utilisation, admission rates for people experiencing homelessness were calculated for the most recent year available (2017/2018) using both observed and MSE-estimated admission numbers. Population denominator estimates for people experiencing homelessness were obtained from ‘The Homelessness Monitor’.^4^ We used the denominator definition for ‘core’ homelessness (rough sleepers, people living in unconventional accommodation, hostels, unsuitable temporary accommodation and sofa surfing). We also conducted a sensitivity analysis restricting the denominator to people sleeping rough or staying in hostels only, which we have named ‘visible’ homelessness to calculate rates. Admission rates were calculated per 1000 population per year. Admission rate ratios were then calculated by comparing the homeless admission rates to the housed population’s admission rate, using data from the HES-APC dataset and published population denominators.^29^ 95% CIs were calculated for the rate ratios.

### Patient and public involvement

This study forms part of SL’s mixed methods PhD project on the preventative role of hospitals for people experiencing homelessness, where Stan Burridge from Expert Focus was the lead patient and public involvement (PPI) advisor. Over 25 people with lived experience of homelessness were involved through workshops and consultations to develop the research priorities, design the studies and review ethical issues. Stan Burridge had a leading role in the qualitative research. Due to the technical nature of this particular study, PPI participants were only involved in the development of the research question and in discussions about the validity of the homelessness phenotype.

## Results

### Observed number of hospital admissions

**Figure 1:**
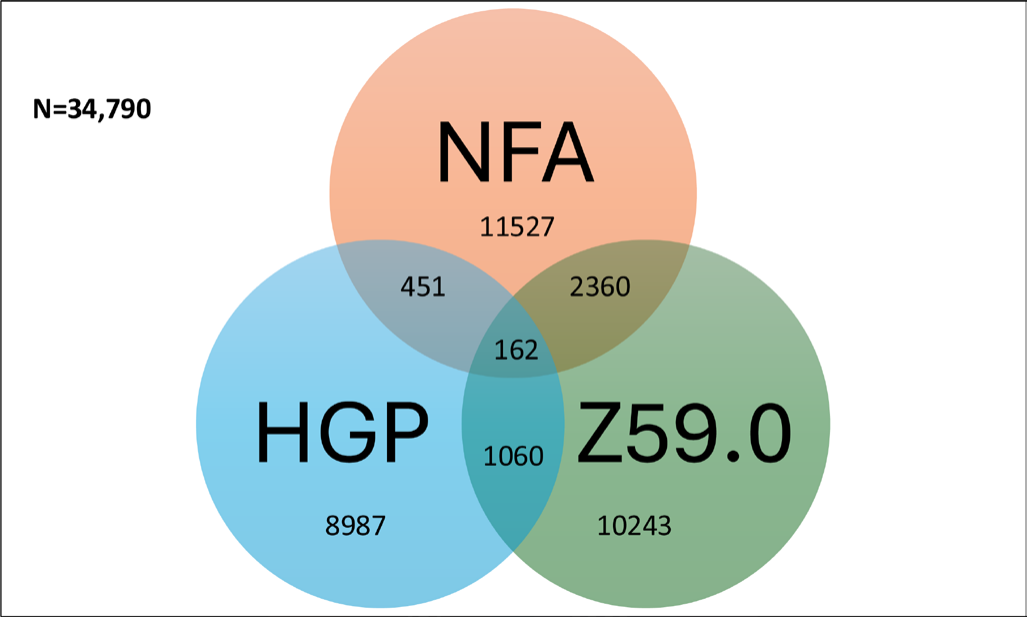
Venn diagram illustrating the observed number of admissions attributed to people experiencing homelessness in England, 2017–2018. The size of the Venn segment is not proportional to the frequency of admissions in that segment. NFA, no fixed abode; HGP, homeless GP; Z59.0, International Classification of Diseases version 10 (ICD-10) code for homelessness.

The total number of admissions where at least one episode of care was coded as homelessness (ie, with one of the three homelessness codes) was 27 124 in 2013/2014, increasing to 31 933 in 2015/16 and 34 790 in 2017/2018. The Venn diagram (figure 1) shows the coding structure of 2017–2018 admission data. Admissions which were singly coded were the most frequent, with NFA having the highest number of coded admissions (n=11 527). Admissions with overlapping homeless codes were relatively infrequent, particularly those coded with all three homeless codes (n=162). Results for other years followed the same overall pattern (see online supplemental table 1).

### Demographics

The age, sex and ethnicity distribution of admissions for people experiencing homelessness compared with the housed population are presented in table 1. People from younger age groups made up the majority of admissions among those experiencing homelessness (46.9% of admissions were for people aged 26–45 years compared with 7.9% for those over 65). In contrast, among the housed population, those from the middle and older age categories were the largest proportion of hospital admissions (admissions for the two oldest categories combined were 72.3%). Nearly three-quarters of admissions for people experiencing homelessness were in men, compared with 44.0% for the housed population. People experiencing homelessness admitted to hospital had a slightly greater proportion of racially minoritised people than those who were housed (white participants comprised 71.7% for people experiencing homelessness and 77.8% for housed people).

**Table 1:** Demographic characteristics of National Health Service (NHS) hospital admissions in England by housing status, 2017/2018

	Homeless N=34 790		Housed N=15 514 367	
	n	%	n	%
Age				
18–25	4349	12.5	985 804	6.4
26–45	16 317	46.9	3 323 951	21.4
46–65	11 376	32.7	4 571 135	29.5
Over 65	2748	7.9	6 633 477	42.8
Sex				
Male	25 362	72.9	6 819 157	44.0
Female	9428	27.1	8 695 210	56.0
Ethnicity				
White (base)	24 944	71.7	12 067 184	77.8
Black/Black British	1983	5.7	444 780	2.9
Asian/Asian British	1600	4.6	785 923	5.1
Mixed	522	1.5	112 953	0.7
Other	1600	4.6	297 953	1.9
Unknown	4140	11.9	1 805 574	11.6

### Multiple systems estimation

The estimated number of unobserved admissions and corresponding BICs based on the eight Poisson models are presented in online supplemental table 2. Model 8 (the full model) had the lowest BIC (80), indicating a superior fit to the data. However, it was not considered for further analysis as it perfectly fitted the data, leaving no df for generalisation and rendering it susceptible to overfitting. Models 5, 6 and 7 also demonstrated low BICs (111, 190 and 102, respectively), but the range of estimates of the unobserved admissions was large and unstable (model 5–86 843 unobserved admissions, model 6–50 030 and model 7–229 697). To mitigate the risk of relying on a single, potentially unstable model, we adopted a model averaging approach. This involved averaging the three best-fitting, non-overfit models (ie, 5,6,7, excluding 8), thereby producing a more robust and reliable overall estimate. The resulting combined total estimate was 176 342 admissions (95% CI 164 031 to 188 654) for 2017/2018.

**Figure 2:**
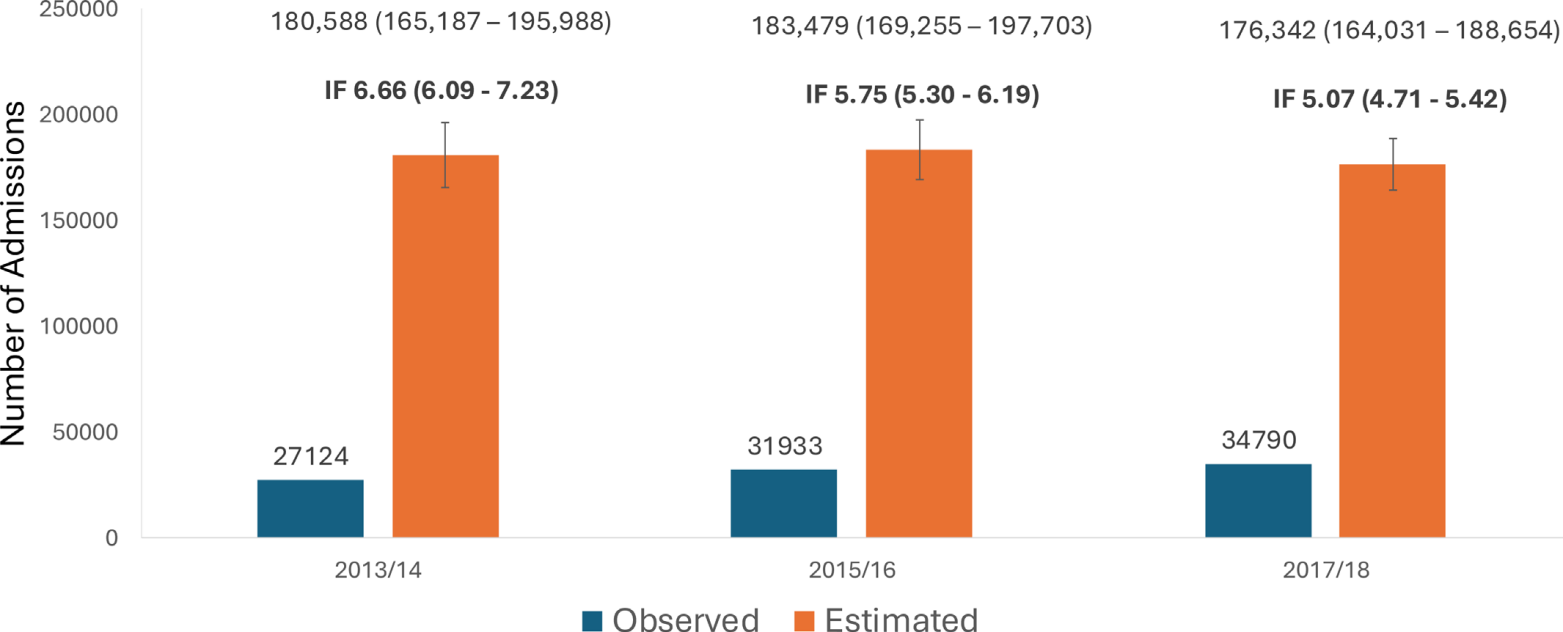
Observed and estimated total number of admissions and inflation factors (IF) by year. Error bars are the 95% CIs around the estimated total. Numerical data are shown within the figure with 95% CIs in brackets. IF, ratio of total estimated admissions to observed admissions.

The average estimated total number of admissions attributed to people experiencing homelessness decreased slightly over time, but the CIs were overlapping (figure 2). The number of observed admissions increased over time and the corresponding inflation factor, the ratio between the estimated total and the observed total, decreased over time. CIs around the inflation factors overlapped between the first and second years of data and the second and third years, but not the first and third. The observed and estimated numbers of admissions, corresponding inflation factors, and 95% CIs for all time periods are presented in figure 2.

### Admission rates and ratios

Based on the observed number of admissions for people experiencing homelessness in 2017/2018 (ie, 34 790 admissions recorded as NFA, HGP or ICD Z59.0), the unadjusted admission rate was 173.4 admissions per 1000 population using the ‘core’ homeless population denominator defined in the Crisis Homeless Monitor^4^ (rough sleepers, people living in unconventional accommodation, hostels, unsuitable temporary accommodation and sofa surfing). As a sensitivity analysis, we restricted the denominator to the ‘visible’ homeless population (rough sleeping and hostel population only) and the unadjusted admission rate was 693.8 admissions per 1000 population. In the housed population, there were 15 514 367 admissions for an estimated 44 168 935 adults in England in 2018,^30^ translating to an unadjusted admission rate of 351.3 admissions per 1000 population per year. compared with the housed population, the admission rate ratios for those recorded as homeless were 0.5 and 2.0 for the core and visible homeless population denominators, respectively (table 2).

**Table 2:** Hospital admission rates for people experiencing homelessness compared with the housed population in England

Numerator	Number of admissions in HES† 2017/2018	Denominator*	Denominator definition and reference	Admission rate per 1000 population per year	Admission rate ratio
Observed admissions for people experiencing homelessness	34 790	200 609	Core homelessness^37^	173.4	0.5
Observed admissions for people experiencing homelessness	34 790	50 144	Visible homelessness^37^	693.8	2.0
Estimated admissions for people experiencing homelessness	176 342 (95% CI 164 031 to 188 654)	200 609	Core homelessness^37^	879.0 (95% CI 817.7 to 940.4)	2.5 (95% CI 2.3 to 2.7)
Estimated admissions for people experiencing homelessness	176 342 (95% CI 164 031 to 188 654)	50 144	Visible homelessness^37^	3516.7 (95% CI 3271.2 to 3762.2)	10.0 (95% CI 9.3 to 10.7)
Housed admissions	15 514 367	44 168 935	General population^30^	351.3	1.0

* There were no CIs provided in estimates of homeless population denominators.

† HES, Hospital Episode Statistics

We estimated that there were 176 342 (95% CI 164 031 to 188 654) admissions in 2017–2018 using MSE, translating to an unadjusted admission rate of 879.0 (95% CI 817.7 to 940.4) admissions per 1000 population per year using the core homeless population denominator. When we restricted to the visible homeless denominator, this resulted in an estimated unadjusted admission rate of 3516.7 (95% CI 3271.2 to 3762.2) admissions per 1000 population per year. We estimated the unadjusted admission rate ratio as 2.5 (95% CI 2.3 to 2.7) for people experiencing homelessness compared with the housed population in England using the core homeless denominator. For the visible homeless population denominator, the unadjusted admission rate ratio was estimated as 10.0 (95% CI 9.3 to 10.7) compared with the housed population (table 2).

## Discussion

This population-based repeated cross-sectional study used national hospital records and MSE to investigate the scale of inpatient activity for people experiencing homelessness in England. We provide a plausible range of admission numbers, rates and ratios to support service planning as well as advocacy for improved data collection on housing status in the English national health service. From an observed 34 790 admissions in 2017/2018, we estimated the total number of homeless admissions to be five times greater at 176,342 (95% CI 164 031 to 188 654). The overall unadjusted admission rate for 2017/2018 using MSE was 879.0 (95% CI 817.7 to 940.4) admissions per 1000 population per year using the ‘core’ homeless population denominator and 3516.7 (95% CI 3271.2 to 3762.2) admissions per 1000 population per year when restricting to the ‘visible’ homeless population. The unadjusted admission rate ratios were estimated as 2.5 (95% CI 2.3 to 2.7) and 10.0 (95% CI 9.3 to 10.7), respectively, compared with the housed adult population of England.

### Findings in the context of the wider literature

Previous research has highlighted the issue of underestimation of homelessness in routine data sources. In England during the COVID-19 pandemic, the ‘Everyone In’ campaign housed 37 000 homeless individuals,^31^ vastly outnumbering the 4266 rough sleepers estimated in 2019 statistics^2^—a clear example of underestimation in official data. Similarly, the Office for National Statistics (ONS) uses MSE methodology to estimate deaths among people experiencing homelessness. In 2018, ONS observed 541 deaths but estimated 726, yielding an inflation factor of 1.34.^32^ Underestimation of homelessness in electronic health data has also been described in other high-income countries, such as the USA^33^,^35^ and Canada.^36^

We compared our findings with two English studies which relied on the NFA code alone to demonstrate the added value of our extended homelessness phenotype for estimating observable (without MSE) inpatient activity. The UK Department of Health’s 2007/2008 assessment of hospital inpatient care for people experiencing homelessness, which identified 17 400 inpatient episodes for 15 800 individuals using only the NFA code. This study did not construct admissions as we have done here, so we used our phenotype to count the observed number of episodes and individuals for comparison. We identified 46 631 episodes for 23 956 individuals in 2017/2018 using the observed data, which is substantially higher than using NFA alone. Similarly, a recent study of emergency admissions of people experiencing homelessness coded with NFA^27^ identified far fewer admissions than our study (14 858 emergency inpatient admissions in 2018/2019). In our study, 82% of our admissions were for an emergency (28 528 admissions in 2017/2018).

The Department of Health study also estimated hospital admission rate ratios for homeless versus housed populations using data from four specialist homeless services in London, Leicester and Cambridge.^13^ Admissions were on average 3.2 times higher for people experiencing homelessness than for the housed population, compared with an admission rate ratio of 2.0 in our study (based on observed admissions relative to ‘visible’ homeless populations (people rough sleeping and living in hostels). However, when we used the CRC estimate as the numerator, we calculated an admission rate ratio of 10.0 (95% CI 9.3 to 10.7) for this population. Our estimates are likely to include a small, but unknown, number of individuals from the broader core homeless population. Consequently, our admission rates for the visibly homeless population may be an overestimate, with rates for the core homeless potentially being an underestimate. Despite this uncertainty, our study provides potential bounded estimates for the admission rate ratio for people experiencing homelessness compared with the general population.

We observed a slight decrease in inflation factors over time, likely due to changes in hospital coding practice which are largely driven by a shifting policy and funding landscape.^37 38^ Online supplemental figure 1 and online supplemental table 1 support this hypothesis, demonstrating an increase in the use of the ICD-10 code for homelessness over time. Although the observed number of homeless admissions rose over time, the total estimated admissions remained stable, leading to a decreasing inflation factor. The stability and relevance of these inflation factors should be confirmed through analyses of additional recent years.

### Strengths

This is the first population-based MSE analysis of hospital admissions for people experiencing homelessness in England and the largest study of this population in the UK to date. For the first time, it quantifies the extent of the significant underestimation of the number of hospital admissions for people experiencing homelessness in routine data. The precise CIs enhance the reliability of the estimates. Our modelling approach accounted for the interdependence of various homeless codes, addressing a common issue in MSE studies. Additionally, the model averaging strategy provided a stable estimate of total admissions and inflation factors. Since the data were collected before the COVID-19 pandemic, they better reflect long-term hospital care utilisation patterns.

### Limitations

MSE can be used to estimate the total number of admissions when accurate data are unavailable, but it cannot attribute outcomes to specific individuals. The validity of the homelessness phenotype used in this study has not been subject to a formal validation study. A recent Canadian validation study demonstrated that the use of the ICD-10 code, Z59.0, which is mandatory in Canada, is highly specific (99.5%), but has lower sensitivity (52.9%). This was likely because people were coded if they had a history of homelessness, rather than current homelessness. It is possible that the codes used in this study have similar issues and that validation and improved coding of housing status is needed.

The original MSE method relies on four key assumptions, as outlined earlier in this paper. Some assumptions were likely violated, yet we consider the overall impact on estimates to be minimal. We assumed our population was sufficiently closed as most individuals do not fully transition in or out of homelessness during a single admission.^39^ The unique identifier, HESID, enabled largely accurate matching between homeless codes when there were multiple episodes of care, although this does not address coding errors.^40^ It is possible that some residual bias was unaccounted for when we adjusted for the lack of independence between homeless codes, which would result in underestimation of the total number of admissions. The greater the overlap between codes, the closer the estimate is to the observed number of admissions.

A key limitation was our inability to adjust for confounders such as age, sex and ethnicity. This arose because we used three different homeless code lists (due to the incomplete nature of homelessness data) which identified very few admissions coded with more than one homelessness code. The low degree of overlap between codes meant our data were too fragmented to create a clear picture of the population size and led to model instability. We used model averaging to address model instability, but because our data were so fragmented, we could only do this for the entire homeless population as a whole. We were, therefore, unable to perform a more detailed analysis that adjusted for important confounders that might influence the likelihood of an admission being coded with one of the homelessness codes and the resulting inflation factor which we calculated. In other words, we may have underestimated or overestimated hospital activity for different subgroups, particularly for more hidden homeless populations such as women.

### Recommendations

In the absence of routine identification and coding of homelessness in healthcare records in England, policy-makers and service providers should consider applying MSE methods to improve estimates of hospital care utilisation for people experiencing homelessness in their specific context. However, we do not advise applying this study’s inflation factors directly as these factors are contingent on context-specific coding practices. For example, areas where there is greater overlap between the homeless codes will likely have better model stability and may, therefore, be able to include covariates, further strengthening the analysis.

Further research should aim to adjust for covariates, particularly age, sex/gender and ethnicity, as these factors vary considerably between homeless and housed populations, as shown in this study. This study’s data predates the pandemic, raising questions about the generalisability of the estimates and inflation factors post-2020. Research is needed to compare these factors during the pandemic, when homelessness decreased due to initiatives like the Everyone In campaign to house all people, and after the pandemic, when rates rose again due to the cost-of-living crisis.^37^

However, a critical step in addressing health inequities in people experiencing homelessness is system-wide uptake and implementation of improved routine coding practices for homelessness. Quite simply, if people are not counted, then they will not count. Researchers have attempted to address this issue through using multiple data sources to identify people experiencing homelessness^33^,^3541^ (although none to our knowledge have applied MSE as we have), using AI and machine learning approaches to identify people,^42^ and through developing bespoke data capture systems.^43^ Making data collection on housing status a routine practice, just like collecting a person’s birthdate or sex/gender, would allow us to more accurately understand how homelessness affects people’s health. While current methods help, they only go so far, and a more standardised approach is needed to capture the full extent of hospital admissions among people experiencing homelessness.

Clinicians should routinely ask about housing status and accurately record it; this would be supported by inclusion of housing status in routine proformas and emphasis of its importance among healthcare providers. Lessons on specific clinical workflows and optimisation of coding practices can be learnt from other contexts.^43^ Similarly, professional coders need specific guidance and training to code homelessness in administrative datasets. For example, Canada’s legal mandate for coding homelessness using the ICD-10 Z59.0 code dramatically increased coding frequency and sensitivity, though specificity needed further improvements.^36^ Although the ICD-10 code is the most specific method for recording homelessness currently available in English hospital records, it fails to capture the diversity of homelessness types, which is linked to different health needs. Moreover, it prevents the calculation of accurate admission rates for a defined population denominator (eg, ‘core’ homeless vs ‘visible’ homeless). Primary care records in England use a wider variety of descriptive codes (eg, for a list of homelessness READ codes and SNOMED codes see^41^), but systematic categorisations of homelessness linked to health risk (such as the EHTOS typology^1^) are also lacking in primary care. National implementation of system-wide improved coding practices is urgently needed to enhance service planning and delivery for people experiencing homelessness.

## Conclusions

We have developed new methods using MSE to identify the scale of hospital admissions for people experiencing homelessness in England. The observed number of homeless admissions increased over the study period, and significant demographic differences were noted between homeless and housed populations. MSE estimates were substantially higher than observed counts, indicating significant underreporting. However, given the methodological concerns, particularly the limited covariate adjustment and potential model instability, the magnitude of this underreporting should be interpreted cautiously. While this study provides valuable insights into the potential scale of hospital admissions among people experiencing homelessness, the methodological limitations necessitate a tempered interpretation of the findings. Future research should prioritise improved identification strategies that allow for covariate adjustment in MSE and ensure model stability. Additionally, further validation of the homelessness phenotype is needed.

## Supplementary material

10.1136/bmjph-2025-002978 online supplemental file 1

## Data Availability

Data may be obtained from a third party and are not publicly available.
