# Supplementary material for: Estimating the scale of hospital admissions for people experiencing homelessness in England: a population-based multiple systems estimation study using national Hospital Episode Statistics
Source: BMJ Public Health. 2025 Oct 28;3(2):e002978. doi: 10.1136/bmjph-2025-002978 (PMC12574433; doi:10.1136/bmjph-2025-002978)
Supplement: online supplemental file 1 [file bmjph-3-2-s001.pdf]

Supplementary Appendix

Estimating the scale of hospital admissions for people experiencing homelessness in England: a population-based multiple systems estimation study using national Hospital Episodes Statistics

Dr Serena Luchenski, PhD[1,2], Prof Dankmar Boehning, PhD[3], Prof Rob Aldridge, PhD[4], Prof Fiona Stevenson, PhD[2], Dr Shema Tariq, PhD[5], and Prof Andrew Hayward, MD[1,2]

1. Collaborative Centre for Inclusion Health, University College London
2. Institute of Epidemiology and Healthcare, University College London
3. S3RI & Mathematical Sciences, University of Southampton
4. Institute for Health Metrics, University of Washington
5. Institute of Global Health, University College London

Corresponding Author: Dr Serena Luchenski, UCL Collaborative Centre for Inclusion Health, 1-19 Torrington Place, London, WC1E 7HT, s.luchenski@ucl.ac.uk

OVERVIEW

In this supplementary appendix, we have included more detailed methods description, additional interim results tables, and Stata statistical code to enable further transparency and reproducibility of this research. This is important because we recommend not merely applying the inflation factors developed in this study to new datasets, but rather to replicate the methods on additional years or different geographies because results will vary depending on how homelessness has been coded.

DETAILED METHODS

Study Design

This is a repeated cross-sectional population-based multiple systems study of hospital admissions for people experiencing homelessness using routine administrative hospital data in England. We have selected this design to account for the underestimation of the number of admissions of people experiencing homelessness in hospital data. We have used the RECORD statement[1] to report this study, which is an extension of STROBE guidelines for reporting of observational epidemiological studies,[2] for routinely collected health data.

Dataset

We used English national Hospital Episodes Statistics (HES) Admitted Patient Care (APC) electronic healthcare records. HES-APC is a hospital activity dataset, rather than an individual-level patient dataset, as its primary purpose is for hospital billing. HES data are provided in a format known as 'Finished Consultant Episodes' (FCEs), representing all the activity that a patient receives under the care of one hospital consultant. Each individual in the dataset has a unique identifier (HESID) which can be used to identify unique individuals and link episodes and admissions for the same individual and to link to other datasets, such as ONS Mortality or other HES datasets, including outpatient care and accident

and emergency (A&E) data. The data can be analysed by FCEs, but for ease of interpretation are often transformed into 'spells' (all of the FCEs for one patient under the care of one hospital provider), 'continuous inpatient spells' (all of the spells contained within one admission, taking into account transfers to different hospital providers that are a part of the same hospital admission), and into individual-level patient activity (in order to examine readmissions). For this study, we focussed on continuous inpatient spells, simply referred to as admissions, for ease of interpretation.

Time Period

We analysed HES-APC data for the years 2013/14, 2015/16, and 2017/18. We selected these years because 2017/18 was the most recent year of data available and the resources available for this project meant that we were only able to analyse three years of data. We left a 1-year gap between time periods to enable us to conduct the analysis over a longer time period, rather than using consecutive years.

Data availability and ethical approval

We worked with researchers at the UCL Institute of Health Informatics who held a full copy of HES for the duration of this study. They provided access to the data, conditional on a data sharing agreement which aligned with the overall programme of work which they had agreed with NHS Digital and obtained ethical approval for. We analysed the data within UCL's secure Data Safe Haven. Ethical approval for this study was granted from the UCL Research Ethics Committee (Ref: 11607/001).

Setting and study population

The setting of this study included all publicly funded NHS inpatient hospitals in England, including acute mental health trusts. The study population of interest was people experiencing homelessness admitted to hospital who were 18 years and older.

As homelessness is not routinely recorded in hospital care records, we consulted people with lived experience of homelessness and clinical collaborators to produce a set of homeless code lists, also known as a homelessness 'phenotype', to identify people experiencing homelessness within HES data. SL has included this phenotype in the publicly available Health Data Research UK (HDRUK) Phenotype Library.[114] The homelessness phenotype includes the following codes:

1. **NFA:** Those whose address is recorded as 'no fixed abode' (NFA), with certain exclusions as per previous research[3] and discussed in the data cleaning section below.

2. **HGP:** Those who were registered at a known homeless GP practice (HGP) that exclusively serves those with homelessness problems. Previous research mapped the names of homeless GP practices in England,[4] but HES only includes alpha-numeric GP practice codes. We used the list of practice names from this research and produced a corresponding list of GP practice codes using the NHS Digital ODS Portal[5] which is freely available and allows users to search for GP practice codes. We then used the practice code list to identify people who were registered at the relevant homeless GP practices within HES.

3. **Z59.0:** Those who have a diagnosis that includes the ICD-10 code for homelessness (Z59.0). If coded, it is likely that this will be coded in one of the secondary diagnosis codes (HES includes up to 20 diagnostic codes per episode of care).

We have included the relevant Stata code, for applying the homelessness phenotype within HES at the end of this appendix.

Outcomes

The primary outcome was the observed and estimated frequencies of hospital admissions for people experiencing homelessness per year in England. Secondary outcomes included the estimated admission rate (number of admissions per 1000 population per year) of people experiencing homelessness in England and the overall admission rate ratio of people experiencing homelessness to housed populations.

Data cleaning

Although HES has undergone cleaning prior to its release by NHS digital, additional cleaning is necessary to produce a sample ready for analysis. We have combined and applied data cleaning rules for the construction of admissions from the University of York Centre for Health Economics[6] and from the seminal Department of Health analysis of people with 'no fixed abode' (NFA)[3] to prepare the dataset. The data cleaning process is detailed in the Stata code below.

Analysis

All statistical analyses and graphs were produced using Stata 17 statistical software. We described the observed hospital inpatient activity for people experiencing homelessness for each year of data using the frequency of admissions overall and by specific homelessness codes (NFA, HGP, or Z59.0). We applied all homeless codes recorded for the episodes attributed to a given admission. For example, if an admission had two episodes, one coded with NFA and one coded with Z59.0, that admission would be coded as having both NFA and Z59.0. Homeless codes are not mutually exclusive and therefore the sum of NFA, HGP or Z59.0 does not equal the total frequency. We produced tables and a Venn Diagram for each year showing the overlap of the frequency (i.e. the duplicates) of admissions coded as NFA, HGP, or Z59.0. We also calculated the number and proportion of people experiencing homelessness and of people who are housed within the adult HES dataset by age, sex, and ethnicity.

Multiple systems estimation (MSE) methods

Previous service evaluation work in London[7] showed that 58-75% of hospital patients known to a specialist homeless health service could be identified using the ICD-10 code for homelessness, registration with a homeless GP, address recorded as NFA, or those whose address was recorded as a known homeless project. To correct for this known under-ascertainment, we developed the phenotype for homelessness within HES-APC data described above and used multiple systems estimation methods (also known as capture recapture methods) [8–10]. We were not able to include addresses for known homeless projects in this analysis because of a lack of address data in our copy of HES-APC (described in the footnotes[1] and limitations).

MSE was initially developed to estimate wildlife population sizes in ecology using two samples/sources. It has been adapted for use in epidemiological studies lacking a population denominator, such as people experiencing homelessness,[11,12] using multiple sources or lists [9,10]. For example, MSE is used by the Office of National Statistics to estimate the number of people who died while homeless in England [11].

[1] We were unable to link homeless project addresses as originally planned because NHS Digital only had capacity to support COVID-specific research during the period when we needed to obtain the data. We therefore used an existing dataset available at UCL, rather than a bespoke linked dataset.

MSE estimates the size of the *total population* from the sum of the population *observed* in two or more sources and by calculating an estimate of the *unobserved* population. The estimated size of the *unobserved* population is based on the number of duplicates in each *observed* source (**Figure 1**). Using this method, we have estimated the total number of admissions for people experiencing homelessness accessing hospitals in inpatient settings by treating each homeless code described above (NFA, HGP, Z59.0) as a ‘source’ to compute MSE estimates.

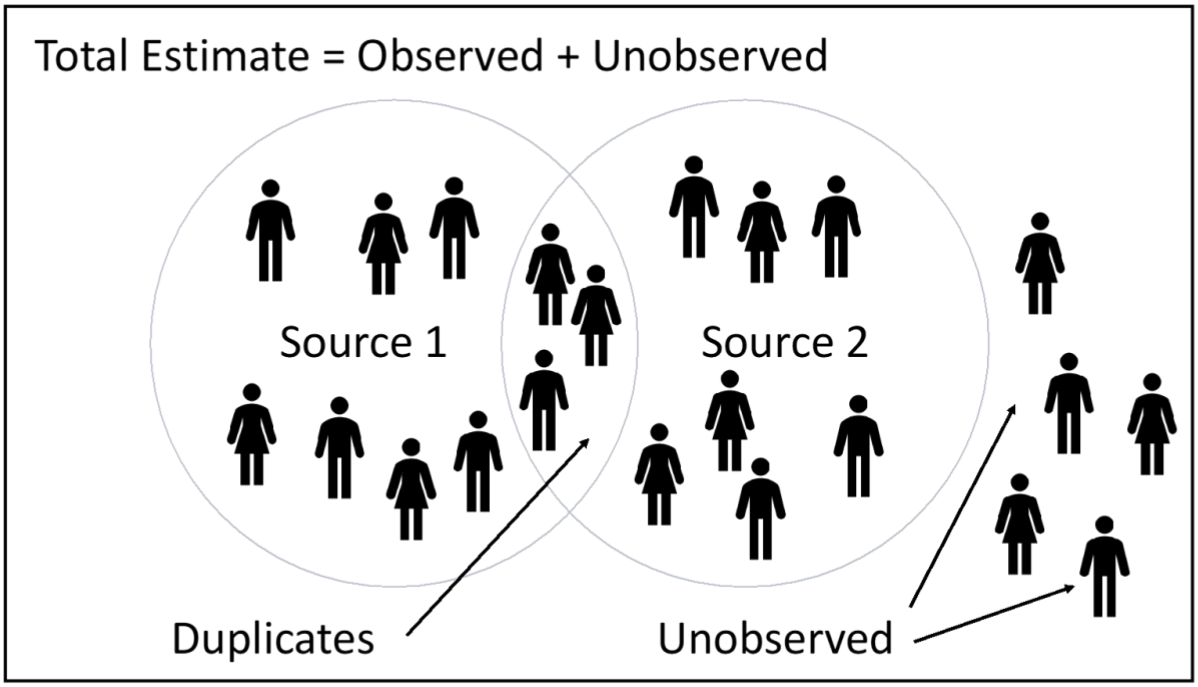

Figure 1. Illustrative example of two-source multiple systems. The unobserved number is estimated statistically based on the number of duplicates in each observed source. The total estimate is the observed plus the unobserved estimate.

The original two source method makes four key assumptions:[9]

1) The population is ‘closed’ (i.e. no in or out migration)
2) Individuals can be matched from capture (source 1) to recapture (source 2)
3) Individuals appearing in one source does not affect individuals appearing in another source (i.e. sources are independent)
4) Capture probabilities are homogenous across all individuals in the population (i.e. individuals are equally likely to be captured, regardless of their characteristics)

In epidemiological studies, assumptions 1 and 2 generally hold if the population is well-defined and the sources are reliably produced.[9,10] This may not always be case, however, for people experiencing homelessness as they may cycle in and out of homelessness and/or give different names or dates of births when encountering different services. In addition, it is nearly always the case that assumptions 3 and 4 are invalid in epidemiological or social research.[9,10] To test and adjust for these assumptions, we have used log linear (Poisson) models to account for these limitations (described below).

Multiple systems estimation of the unobserved population

We used the number of admissions in each of the three 'sources' (NFA, HGP, Z59.0) and the overlap between each source to compute MSE estimates. We produced eight Poisson models to estimate the total number of episodes for people experiencing homelessness (Model 1 – include counts of each source as main effects only, Models 2-4 include main effects and all individual two-way interactions, Models 5-7 include main effects and two two-way interactions, and Model 8 which is the full model and includes main effects and all three two-way interaction terms). For example, model 2 is Log(count) = intercept + a NFA + b HGP + c Z59.0 + (ab) NFA_HGP. Details are found in Table 2. After each model is fitted, Stata can estimate the size of the unobserved population and its standard error using the 'predict' command.

We planned to judge each model according to the Bayesian Information Criterion (BIC) and goodness of fit tests. However, when the models were fitted there was a wide range of estimates produced for the unobserved population for the best models. Based on previous methodological research[10], we conducted Bayesian model averaging. This involved calculating weighted averages of the three best models (i.e. those three with the lowest BIC) to produce more stable estimates of the total population and corresponding inflation factors.

To calculate the weights for model averaging, suppose there are J models with total population sizes $N_1,\ldots,N_J$ as well as BIC values $B_1,\ldots,B_J$. Weights are computed according to:

$$W_i=\exp(-B_i)$$

and the weighted average is then given by

$$N_{ave} = (W_1*N_1+\ldots + W_J*N_J)/(W_1+\ldots.W_J)$$

All BIC values were scaled down first by dividing each of them by 100 because exp(-large number) can lead to numerical instabilities. We also applied the same BIC averaging approach to the standard errors of the estimates and then computed a final 95% confidence interval based on the normal distribution (i.e. 1.96 +/- standard error) around the final model averaged estimate.

Correcting for under ascertainment of hospital inpatient activity

The *total* is estimated by adding the observed and unobserved estimate together. We then used the MSE *total* estimate and divided it by the size of the *observed* estimate to develop an inflation factor for estimating the true size of homeless in-patient activity. For example, if MSE estimated that only 50% of admissions for people experiencing homelessness were *observed*, this would give an inflation factor of 2. Therefore, the estimated counts of hospital admissions would be doubled. We computed the same calculation for the point estimate and the upper and lower confidence limits. We have presented results graphically to visualise the observed and inflated estimates across the three different years of data.

Independence

To test for the assumption of independence of sources, we examined the correlation structure between each of the three 'sources' (i.e. homeless codes) using the 2017/18 admissions data. We hypothesised that NFA and HGP would be negatively correlated (i.e. patients would be more likely to give an address if they were registered with a homeless GP) and NFA and Z59.0 would be positively correlated (i.e. patients would be more likely to be coded as Z59.0 if their address was categorised as NFA as clinicians would be more likely to know patients were experiencing homelessness). We did not hypothesise any particular association between HGP and Z59.0. Using, the full model (i.e. with all main effects and all three two-way interactions), we calculated the odds ratios and 95% confidence intervals for a person being coded with:

NFA and HGP, given they were also coded with Z59.0
NFA and Z59.0, given they were also coded with HGP
HGP and Z59.0, given they were also coded with NFA

An OR of 1.0 would indicate the sources were independent.

Homogeneity

The assumption of homogeneity means that the probability of being coded with one homeless code is constant for all individuals within a given source, regardless of the characteristics of those individuals. As in all epidemiological studies, it is reasonable to assume that covariates such as age, sex, and ethnicity are important confounders to include in the models. However, this needs to be balanced computationally. As can be seen in the results below, the number of duplicates in each source was not very high (11.6% of admissions were duplicates in two sources, 0.5% were duplicates in all three sources). If we had further split the data by demographic factors, this would have created further numerical instability in our estimates. We have discussed the implications of this within the limitations of this study.

Estimating admission rates and admission rate ratios

We have estimated admission rates for people experiencing homelessness for 2017/18 (our most recent year of data available) using the *observed* number of admissions and the MSE estimates of the *total* number of admissions produced in this study and homeless population denominator estimates from the literature.[13] We have then compared these admission rates to the admission rate of the housed population in our HES-APC dataset and compared to previous research in the discussion.

As discussed previously, homelessness is not routinely recorded in official datasets. It is not possible to know for certain what the true underlying population is in this study because of the lack of coding of specific types of homelessness within HES data. We have used two estimated population denominators of the number of people experiencing homelessness in England published by Fitzpatrick and colleagues in The Homelessness Monitor[14] to calculate a range of plausible admission rates. These include 'core' and 'visible' people experiencing homelessness, which were the best available estimates at the time of writing.

From 2018-19, the Crisis Homeless Monitor began estimating the number of people experiencing 'core' homelessness, which includes rough sleepers, people living in unconventional accommodation (e.g. squatting), hostels, unsuitable temporary accommodation (e.g. bed and breakfasts), and sofa surfing, given the limitations of official routine statistics.[14] There were an estimated 200,609 people experiencing core homelessness in England in 2018-19,[14] which is the closest year available to the data we have analysed in this study at the time of writing. The other relevant population denominator is to restrict the core homelessness population to the most 'visible' forms of homelessness, which is the rough sleeping and hostel populations, estimated as 50,144 people for the same year.[14] The 'core' homeless denominator is the most inclusive definition and was expected to result in the most conservative estimates of admission rates, whereas the 'visible' homeless definition was expected to yield the highest admission rates, but to be more comparable to studies which have focussed on rough sleeper and hostel populations.

We estimated admission rates by dividing the number of admissions for each year by the population denominator estimate and multiplying this by 1000 to give a standardised rate per 1000 population per year. We have not adjusted for any confounding factors, such as age, sex, and ethnicity, because of the lack of age-, sex-, and ethnicity- specific denominator data. We compared the admission rates for people

experiencing homelessness (1-4, below) to the rates in the general housed population (5, below) using admission rate ratios with 95% confidence intervals.

1) Admission rate of the *observed* number of admissions for people experiencing homelessness in 2017/18 relative to the *core homeless* population in 2018/19;
2) Admission rate of the *observed* number of admissions for people experiencing homelessness in 2017/18 relative to the *visible homeless* population in 2018/19;
3) Admission rate of the MSE *estimated total* number of admissions for people experiencing homelessness in 2017/18 relative to the *core homeless* population in 2018/19;
4) Admission rate of the MSE *estimated total* number of admissions for people experiencing homelessness in 2017/18 relative to the *visible homeless* population in 2018/19; and
5) Admission rate of the number of admissions for people who are *housed* in 2017/18.

Results

In addition to the results presented in the main paper, we have included several intermediary figures and tables to help readers understand more about the nature of the data to support their own future analyses. These include a figure showing the frequency of admissions by homelessness code and year (supplementary figure 1) – the main paper includes a Venn diagram for 2017/18; the frequency dataset used for computing the multiple systems estimates (supplementary table 1); and the detailed multiple systems estimates of admissions for each regression model (supplementary table 2).

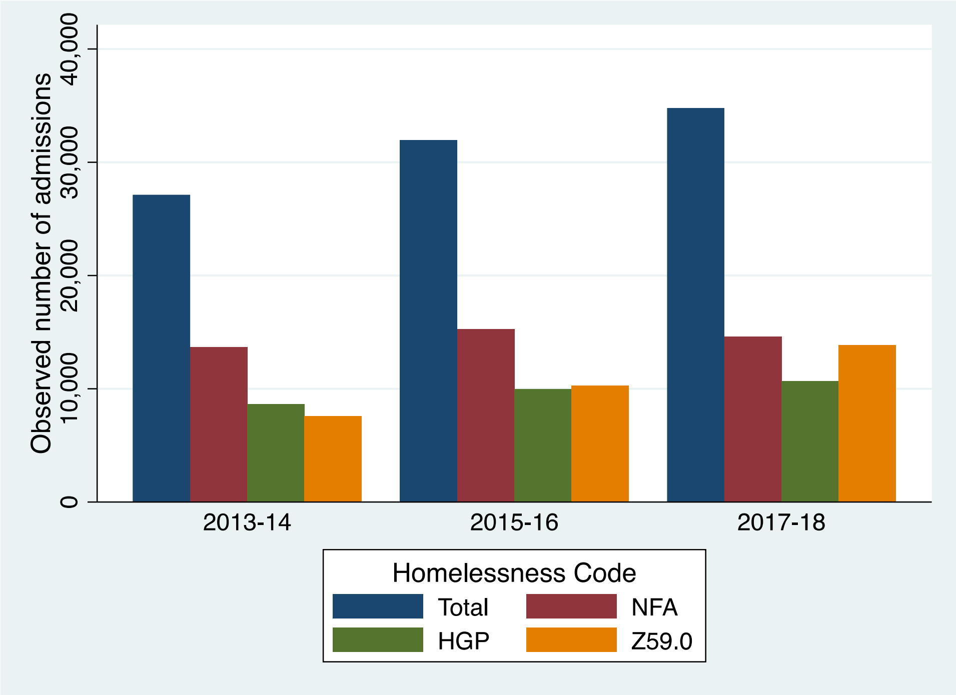

Supplementary Figure 1. Frequency of admissions for people experiencing homelessness in England by homelessness code and year, 2013-18. NFA = 'no fixed abode', HGP = 'homeless GP', Z59.0 = ICD-10 code for homelessness, total = being coded with at least one of the three homeless codes.

Supplementary Table 1. Dataset for computing multiple systems estimates of 2017/18 HES admissions.

	Main Effects			Interaction Terms			Observed Data Frequency		
Model	NFA	HGP	Z59.0	NFA_HGP	NFA_Z59.0	HGP_ICD	2013/14 n = 27,124	2015/16 n = 31,933	2017/18 n = 34,790
1	1	0	0	0	0	0	11445	12628	11527
2	0	1	0	0	0	0	7673	8637	8987
3	0	0	1	0	0	0	5444	7290	10243
4	1	1	0	1	0	0	435	408	451
5	1	0	1	0	1	0	1621	2033	2360
6	0	1	1	0	0	1	382	805	1060
7	1	1	1	1	1	1	124	132	162
8	0	0	0	0	0	0	.	.	.

Supplementary Table 2. Multiple systems estimates of unobserved and total admissions. Total estimated admissions = unobserved + observed admissions, where observed = 27,124 admissions for 2013/14, 31,933 for 2015/16, and 34,790 for 2017/18.

		2013/14			2015/16			2017/18		
Model	Interaction Terms	BIC	Unobserved	Total	BIC	Unobserved	Total	BIC	Unobserved	Total
1	None	1190	76050	103174	1366	80980	112913	1126	80,557	115,347
2	NFA_HGP	524	50045	77169	303	53197	85130	339	59,951	94,741
3	NFA_Z59.0	164	150932	178056	490	140960	172893	385	129,621	164,411
4	HGP_ Z59.0	1153	70870	97994	1366	82119	114052	1123	78,669	113,459
5	NFA_HGP, NFA_ Z59.0	115	109350	136474	118	78216	110149	111	86,843	121,633
6	NFA_HGP, HGP_ Z59.0	260	38437	65561	190	45282	77215	190	50,030	84,820
7	NFA_ Z59.0, HGP_ Z59.0	76	201879	229003	110	267324	299257	102	229,697	264,487
8	NFA_HGP, NFA_ Z59.0, HGP_ Z59.0	78	220082	247206	79	157183	189116	80	152,363	187,153

Stata Code

1. MSE (Capture Recapture) Dataset Creation

```
/************************

CREATE DATSETS FOR CONDUCTING MSE ANALYSIS

************************/

*2013/14 Data

clear
use "S:\HLE_HES_Luchenski\HLE HES Oct 22 data\APC\APC_2013_CLEAN.dta"

* This code would only keep episodes labelled as homeless
keep if nfa == 1 | gpbin ==1 | z590 ==1
count

save "S:\HLE_HES_Luchenski\HLE HES Oct 22 data\APC\APC_2013_CLEAN_CRC.dta", replace


capture log using CRC_2013, replace


/*Generate Variables to Count for Venn*/
capture gen venn1 =0
replace venn1 =1 if nfa ==1 & gpbin != 1 & z590 !=1
capture gen venn2 =0
replace venn2 =1 if gpbin ==1 & nfa != 1 & z590 !=1
capture gen venn3 =0
```

```
replace venn3 =1 if z590 ==1 & nfa != 1 & gpbin !=1
capture gen venn4 =0
replace venn4 =1 if nfa ==1 & gpbin == 1 & z590 !=1
capture gen venn5 =0
replace venn5 =1 if nfa ==1 & gpbin != 1 & z590 ==1
capture gen venn6 =0
replace venn6 =1 if nfa !=1 & gpbin == 1 & z590 ==1
capture gen venn7 =0
replace venn7 =1 if nfa ==1 & gpbin == 1 & z590 ==1
*/

/*Using the individual venn variables above led to small errors in the admissions/individuals numbers
Have created an overall venn variable which has resolved the issue with the numbers*/

gen fullvenn = 0
replace fullvenn = 1 if venn1 ==1
replace fullvenn = 2 if venn2 ==1
replace fullvenn = 3 if venn3 ==1
replace fullvenn = 4 if venn4 ==1
replace fullvenn = 5 if venn5 ==1
replace fullvenn = 6 if venn6 ==1
replace fullvenn = 7 if venn7 ==1

tab fullvenn, miss

tabout fullvenn using CRC_data.text, oneway replace



*Episodes
foreach w in nfa gpbin z590 fullvenn {
```

```
tabout `w' using CRC_data.text, oneway append
}

*Admissions
foreach w in nfa gpbin z590 fullvenn {
preserve
collapse (max) `w', by(CHE_CIPS)
tabout `w' using CRC_data.text, oneway append
restore
}

*Patients
foreach w in nfa gpbin z590 fullvenn {
preserve
collapse (max) `w', by(encrypted_hesid)
tabout `w' using CRC_data.text, oneway append
restore
}

log close


****************************************************************************

*2015/16 Data

clear
use "S:\HLE_HES_Luchenski\HLE HES Oct 22 data\APC\APC_2015_CLEAN.dta"

* This code would only keep episodes labelled as homeless
```

```
keep if nfa == 1 | gpbin ==1 | z590 ==1
count

save "S:\HLE_HES_Luchenski\HLE HES Oct 22 data\APC\APC_2015_CLEAN_CRC.dta", replace

capture log using CRC_2015, replace


/*Generate Variables to Count for Venn*/
capture gen venn1 =0
replace venn1 =1 if nfa ==1 & gpbin != 1 & z590 !=1
capture gen venn2 =0
replace venn2 =1 if gpbin ==1 & nfa != 1 & z590 !=1
capture gen venn3 =0
replace venn3 =1 if z590 ==1 & nfa != 1 & gpbin !=1
capture gen venn4 =0
replace venn4 =1 if nfa ==1 & gpbin == 1 & z590 !=1
capture gen venn5 =0
replace venn5 =1 if nfa ==1 & gpbin != 1 & z590 ==1
capture gen venn6 =0
replace venn6 =1 if nfa !=1 & gpbin == 1 & z590 ==1
capture gen venn7 =0
replace venn7 =1 if nfa ==1 & gpbin == 1 & z590 ==1
*/

/*Using the individual venn variables above led to small errors in the admissions/individuals numbers
Have created an overall venn variable which has resolved the issue with the numbers*/

gen fullvenn = 0
replace fullvenn = 1 if venn1 ==1
```

```
replace fullvenn = 2 if venn2 ==1
replace fullvenn = 3 if venn3 ==1
replace fullvenn = 4 if venn4 ==1
replace fullvenn = 5 if venn5 ==1
replace fullvenn = 6 if venn6 ==1
replace fullvenn = 7 if venn7 ==1

tab fullvenn, miss


*Episodes
foreach w in nfa gpbin z590 fullvenn {
tabout `w' using CRC_data.text, oneway append
}

*Admissions
foreach w in nfa gpbin z590 fullvenn {
preserve
collapse (max) `w', by(CHE_CIPS)
tabout `w' using CRC_data.text, oneway append
restore
}

*Patients
foreach w in nfa gpbin z590 fullvenn {
preserve
collapse (max) `w', by(encrypted_hesid)
tabout `w' using CRC_data.text, oneway append
restore
}
```

```
log close

***************************************************************************

*2017/18 Data

clear
use "S:\HLE_HES_Luchenski\HLE HES Oct 22 data\APC\APC_2017_CLEAN.dta"

* This code would only keep episodes labelled as homeless
keep if nfa == 1 | gpbin ==1 | z590 ==1
count

save "S:\HLE_HES_Luchenski\HLE HES Oct 22 data\APC\APC_2017_CLEAN_CRC.dta", replace

capture log using CRC_2017, replace

/*Generate Variables to Count for Venn*/
capture gen venn1 =0
replace venn1 =1 if nfa ==1 & gpbin != 1 & z590 !=1
capture gen venn2 =0
replace venn2 =1 if gpbin ==1 & nfa != 1 & z590 !=1
capture gen venn3 =0
replace venn3 =1 if z590 ==1 & nfa != 1 & gpbin !=1
capture gen venn4 =0
replace venn4 =1 if nfa ==1 & gpbin == 1 & z590 !=1
capture gen venn5 =0
replace venn5 =1 if nfa ==1 & gpbin != 1 & z590 ==1
capture gen venn6 =0
```

```
replace venn6 =1 if nfa !=1 & gpbin == 1 & z590 ==1
capture gen venn7 =0
replace venn7 =1 if nfa ==1 & gpbin == 1 & z590 ==1
*/

/*Using the individual venn variables above led to small errors in the admissions/individuals numbers
Have created an overall venn variable which has resolved the issue with the numbers*/

gen fullvenn = 0
replace fullvenn = 1 if venn1 ==1
replace fullvenn = 2 if venn2 ==1
replace fullvenn = 3 if venn3 ==1
replace fullvenn = 4 if venn4 ==1
replace fullvenn = 5 if venn5 ==1
replace fullvenn = 6 if venn6 ==1
replace fullvenn = 7 if venn7 ==1

tab fullvenn, miss



*Episodes
foreach w in nfa gpbin z590 fullvenn {
tabout `w' using CRC_data.text, oneway append
}

*Admissions
foreach w in nfa gpbin z590 fullvenn {
preserve
collapse (max) `w', by(CHE_CIPS)
tabout `w' using CRC_data.text, oneway append
```

```
restore
}

*Patients
foreach w in nfa gpbin z590 fullvenn {
preserve
collapse (max) `w', by(encrypted_hesid)
tabout `w' using CRC_data.text, oneway append
restore
}
log close
```

The Dataset will look like this:

**model**	**nfa**	**hgp**	**icd**	**nfa_hgp**	**nfa_icd**	**hgp_icd**	**epis13**	**epis15**	**epis17**	**adm13**	**adm15**	**adm17**	**indiv13**	**indiv15**	**indiv17**
1	1	0	0	0	0	0	14079	15624	14692	11445	12628	11527	7764	8704	7985
2	0	1	0	0	0	0	9465	11059	11442	7673	8637	8987	3594	4123	4866
3	0	0	1	0	0	0	7411	10200	14831	5444	7290	10243	4297	5729	8112
4	1	1	0	1	0	0	570	560	632	435	408	451	169	181	213
5	1	0	1	0	1	0	2087	2766	3282	1621	2033	2360	1244	1507	1837
6	0	1	1	0	0	1	531	1102	1521	382	805	1060	268	456	821
7	1	1	1	1	1	1	166	176	231	124	132	162	83	106	122
8	0	0	0	0	0	0									

2. CRC Analysis – Poisson Regression

```
******************************************************
* Author:                 Serena Luchenski
```

```
* Date Created:                    30/11/2022
* Purpose:                         Conduct CRC analysis using three years  of HES APC Data

*****************************************************

clear
use "S:\HLE_HES_Luchenski\CRC_Files\crc_data_dec_22.dta"
set more off
capture log using homeless_CRC, replace



*Use Poisson Regression to Calculate Estimates

********************2013/14************************

*  Main effects only
foreach w in epis13 adm13 indiv13  {
        poisson `w' nfa hgp icd
        estat ic
        eststo ic
        estat gof
        scalar gof = r(chi2_p)
        estadd scalar gofchi2 = gof:ic
        estout ic using crc_bic_gof.text, cells(none) stats(bic gofchi2) mlabels(m1`w') append
        capture predict n1`w', n
        }

* Individual two-way interactions
foreach w in epis13 adm13 indiv13  {
        poisson `w' nfa hgp icd  nfa_hgp
```

```
        estat ic
        eststo ic
        estat gof
        scalar gof = r(chi2_p)
        estadd scalar gofchi2 = gof:ic
        estout ic using crc_bic_gof.text, cells(none) stats(bic gofchi2) mlabels(m2`w') append
        capture predict n2`w', n
        }

foreach w in epis13 adm13 indiv13  {
        poisson `w' nfa hgp icd  nfa_icd
        estat ic
        eststo ic
        estat gof
        scalar gof = r(chi2_p)
        estadd scalar gofchi2 = gof:ic
        estout ic using crc_bic_gof.text, cells(none) stats(bic gofchi2) mlabels(m3`w') append
        capture predict n3`w', n
        }

foreach w in epis13 adm13 indiv13  {
        poisson `w' nfa hgp icd  hgp_icd
        estat ic
        eststo ic
        estat gof
        scalar gof = r(chi2_p)
        estadd scalar gofchi2 = gof:ic
        estout ic using crc_bic_gof.text, cells(none) stats(bic gofchi2) mlabels(m4`w') append
        capture predict n4`w', n
        }
```

```
* Two two-way interactions
foreach w in epis13 adm13 indiv13  {
	poisson `w' nfa hgp icd  nfa_hgp nfa_icd
	estat ic
	eststo ic
	estat gof
	scalar gof = r(chi2_p)
	estadd scalar gofchi2 = gof:ic
	estout ic using crc_bic_gof.text, cells(none) stats(bic gofchi2) mlabels(m5`w') append
	capture predict n5`w', n
	}

foreach w in epis13 adm13 indiv13 {
	poisson `w' nfa hgp icd  nfa_hgp hgp_icd
	estat ic
	eststo ic
	estat gof
	scalar gof = r(chi2_p)
	estadd scalar gofchi2 = gof:ic
	estout ic using crc_bic_gof.text, cells(none) stats(bic gofchi2) mlabels(m6`w') append
	capture predict n6`w', n
	}

foreach w in epis13 adm13 indiv13 {
	poisson `w' nfa hgp icd  nfa_icd hgp_icd
	estat ic
	eststo ic
	estat gof
	scalar gof = r(chi2_p)
```

```
	estadd scalar gofchi2 = gof:ic
	estout ic using crc_bic_gof.text, cells(none) stats(bic gofchi2) mlabels(m7`w') append
	capture predict n7`w', n
	}

*Full model
foreach w in epis13 adm13 indiv13 {
	poisson `w' nfa hgp icd nfa_hgp nfa_icd hgp_icd
	estat ic
	eststo ic
	estat gof
	scalar gof = r(chi2_p)
	estadd scalar gofchi2 = gof:ic
	estout ic using crc_bic_gof.text, cells(none) stats(bic gofchi2) mlabels(m8`w') append
	capture predict n8`w', n
	}


*******************2015/16************************

*  Main effects only
foreach w in epis15 adm15 indiv15 {
	poisson `w' nfa hgp icd
	estat ic
	eststo ic
	estat gof
	scalar gof = r(chi2_p)
	estadd scalar gofchi2 = gof:ic
	estout ic using crc_bic_gof.text, cells(none) stats(bic gofchi2) mlabels(m1`w') append
	capture predict n1`w', n
```

```
}

* Individual two-way interactions
foreach w in epis15 adm15 indiv15 {
	poisson `w' nfa hgp icd  nfa_hgp
	estat ic
	eststo ic
	estat gof
	scalar gof = r(chi2_p)
	estadd scalar gofchi2 = gof:ic
	estout ic using crc_bic_gof.text, cells(none) stats(bic gofchi2) mlabels(m2`w') append
	capture predict n2`w', n
	}

foreach w in epis15 adm15 indiv15 {
	poisson `w' nfa hgp icd  nfa_icd
	estat ic
	eststo ic
	estat gof
	scalar gof = r(chi2_p)
	estadd scalar gofchi2 = gof:ic
	estout ic using crc_bic_gof.text, cells(none) stats(bic gofchi2) mlabels(m3`w') append
	capture predict n3`w', n
	}

foreach w in epis15 adm15 indiv15 {
	poisson `w' nfa hgp icd  hgp_icd
estat ic
	eststo ic
	estat gof
```

```
            scalar gof = r(chi2_p)
            estadd scalar gofchi2 = gof:ic
            estout ic using crc_bic_gof.text, cells(none) stats(bic gofchi2) mlabels(m4`w') append
            capture predict n4`w', n
            }

* Two two-way interactions
foreach w in epis15 adm15 indiv15 {
            poisson `w' nfa hgp icd  nfa_hgp nfa_icd
            estat ic
            eststo ic
            estat gof
            scalar gof = r(chi2_p)
            estadd scalar gofchi2 = gof:ic
            estout ic using crc_bic_gof.text, cells(none) stats(bic gofchi2) mlabels(m5`w') append
            capture predict n5`w', n
            }

foreach w in epis15 adm15 indiv15 {
            poisson `w' nfa hgp icd  nfa_hgp hgp_icd
            estat ic
            eststo ic
            estat gof
            scalar gof = r(chi2_p)
            estadd scalar gofchi2 = gof:ic
            estout ic using crc_bic_gof.text, cells(none) stats(bic gofchi2) mlabels(m6`w') append
            capture predict n6`w', n
            }

foreach w in epis15 adm15 indiv15 {
```

```
	poisson `w' nfa hgp icd  nfa_icd hgp_icd
	estat ic
	eststo ic
	estat gof
	scalar gof = r(chi2_p)
	estadd scalar gofchi2 = gof:ic
	estout ic using crc_bic_gof.text, cells(none) stats(bic gofchi2) mlabels(m7`w') append
	capture predict n7`w', n
	}

*Full model
foreach w in epis15 adm15 indiv15 {
	poisson `w' nfa hgp icd nfa_hgp nfa_icd hgp_icd
	estat ic
	eststo ic
	estat gof
	scalar gof = r(chi2_p)
	estadd scalar gofchi2 = gof:ic
	estout ic using crc_bic_gof.text, cells(none) stats(bic gofchi2) mlabels(m8`w') append
	capture predict n8`w', n
	}



*******************2017/18******************

*  Main effects only
foreach w in epis17 adm17 indiv17 {
	poisson `w' nfa hgp icd
	estat ic
```

```
	eststo ic
	estat gof
	scalar gof = r(chi2_p)
	estadd scalar gofchi2 = gof:ic
	estout ic using crc_bic_gof.text, cells(none) stats(bic gofchi2) mlabels(m1`w') append
	capture predict n1`w', n
	}

* Individual two-way interactions
foreach w in epis17 adm17 indiv17 {
	poisson `w' nfa hgp icd  nfa_hgp
	estat ic
	eststo ic
	estat gof
	scalar gof = r(chi2_p)
	estadd scalar gofchi2 = gof:ic
	estout ic using crc_bic_gof.text, cells(none) stats(bic gofchi2) mlabels(m2`w') append
	capture predict n2`w', n
	}

foreach w in epis17 adm17 indiv17 {
	poisson `w' nfa hgp icd  nfa_icd
	estat ic
	eststo ic
	estat gof
	scalar gof = r(chi2_p)
	estadd scalar gofchi2 = gof:ic
	estout ic using crc_bic_gof.text, cells(none) stats(bic gofchi2) mlabels(m3`w') append
	capture predict n3`w', n
	}
```

```
foreach w in epis17 adm17 indiv17 {
	poisson `w' nfa hgp icd  hgp_icd
	estat ic
	eststo ic
	estat gof
	scalar gof = r(chi2_p)
	estadd scalar gofchi2 = gof:ic
	estout ic using crc_bic_gof.text, cells(none) stats(bic gofchi2) mlabels(m4`w') append
	capture predict n4`w', n
	}

* Two two-way interactions
foreach w in epis17 adm17 indiv17 {
	poisson `w' nfa hgp icd  nfa_hgp nfa_icd
	estat ic
	eststo ic
	estat gof
	scalar gof = r(chi2_p)
	estadd scalar gofchi2 = gof:ic
	estout ic using crc_bic_gof.text, cells(none) stats(bic gofchi2) mlabels(m5`w') append
	capture predict n5`w', n
	}

foreach w in epis17 adm17 indiv17 {
	poisson `w' nfa hgp icd  nfa_hgp hgp_icd
	estat ic
	eststo ic
	estat gof
	scalar gof = r(chi2_p)
```

```
    estadd scalar gofchi2 = gof:ic
    estout ic using crc_bic_gof.text, cells(none) stats(bic gofchi2) mlabels(m6`w') append
    capture predict n6`w', n
    }

foreach w in epis17 adm17 indiv17 {
    poisson `w' nfa hgp icd  nfa_icd hgp_icd
    estat ic
    eststo ic
    estat gof
    scalar gof = r(chi2_p)
    estadd scalar gofchi2 = gof:ic
    estout ic using crc_bic_gof.text, cells(none) stats(bic gofchi2) mlabels(m7`w') append
    capture predict n7`w', n
    }
*Full model
foreach w in epis17 adm17 indiv17 {
    capture poisson `w' nfa hgp icd nfa_hgp nfa_icd hgp_icd
    estat ic
    eststo ic
    estat gof
    scalar gof = r(chi2_p)
    estadd scalar gofchi2 = gof:ic
    estout ic using crc_bic_gof.text, cells(none) stats(bic gofchi2) mlabels(m8`w') append
    capture predict n8`w', n
    }

*** Export dataset to get predicted variables ****
export excel using "S:\HLE_HES_Luchenski\CRC_Files\CRC_predict.xlsx", firstrow(variables) replace
```

log close

Example of Results Table:

Admissions		2013/14	Observed n =	27124		
**Model**	**Interaction Terms**	**BIC**	**Pearson's GOF**	**Unobserved**	**Total**	**Inflation Factor**
1	none	1190	1113	76050	103174	3.8
2	nfa_hgp	524	430	50045	77169	2.8
3	nfa_icd	164	93	150932	178056	6.6
4	hgp_icd	1153	1024	70870	97994	3.6
5	nfa_hgp, nfa_icd	115	46	109350	136474	5.0
6	nfa_hgp, hgp_icd	260	288	38437	65561	2.4
7	nfa_icd, hgp_icd	76	1	201879	229003	8.4
8	nfa_hgp, nfa_icd, hgp_icd	78	0	220082	247206	9.1

REFERENCES

1 Benchimol EI, Smeeth L, Guttmann A, *et al.* The REporting of studies Conducted using Observational Routinely-collected health Data (RECORD) Statement. *PLoS Med*. 2015;12. doi: 10.1371/journal.pmed.1001885

2 von Elm E, Altman DG, Egger M, *et al.* Strengthening the Reporting of Observational Studies in Epidemiology (STROBE) statement: guidelines for reporting observational studies. *BMJ*. 2007;335:806–8. doi: 10.1136/bmj.39335.541782.AD

3 Department of Health. Healthcare for single homeless people. London 2010.

4 Crane M, Cetrano G, Joly L, *et al.* Mapping of Specialist Primary Health Care Services. 2018.

5 NHS DIgital. NHS Digital ODS Portal. 2024. https://odsportal.digital.nhs.uk/ (accessed 10 October 2024)

6 Arabadzhyan A, Castelli A, Gaughan J, *et al.* A comparison of NHS Digital and CHE algorithms for spells and CIPS construction: Supplementary material to CHE Research Paper 182 Productivity of the English National Health Service 2018/19 update. York 2021.

7 King's Health Partners Pathway Team. KHP Pathway Homeless Team - First Year Report 2014. 2015.

8 Jensen RT, Pearson M. Generating Data on "Hard-to-Count" Populations: A Field Guide to the Rapid Assessment Plus (RA+) Methodology. Cambridge, Massachusetts 2001.

9 IWGDM. Capture-recapture and multiple-record systems estimation I: History and theoretical development. International Working Group for Disease Monitoring and Forecasting. *Am J Epidemiol*. 1995;142:1047–58.

10 Hook EB, Regal RR. Capture-Recapture Methods in Epidemiology: Methods and Limitations. *Epidemiol Rev*. 1995;17:243–64. doi: 10.1093/oxfordjournals.epirev.a036192

11 Office for National Statistics. Deaths of homeless people in England and Wales: 2013 to 2017. 2018.

12 Fisher N, Turner SW, Pugh R, *et al.* Estimating numbers of homeless and homeless mentally ill people in north east Westminster by using capture-recapture analysis. *BMJ*. 1994;308:27–30.

13 Fitzpatrick S, Bramley G, McMordie L, *et al.* The Homelessness Monitor: England 2023 . London 2023.

14 Fitzpatrick S, Pawson H, Bramley G, *et al.* The Homelessness Monitor: England. London 2021.
